# Industrial-Scale Production and Applications of Bacterial Cellulose

**DOI:** 10.3389/fbioe.2020.605374

**Published:** 2020-12-22

**Authors:** Chunyan Zhong

**Affiliations:** Hainan Yeguo Foods Co. Ltd., Hainan, China

**Keywords:** bacterial cellulose, nanofiber, industrial fermentation, commercial applications, three-dimensional reticulated network

## Abstract

Bacterial cellulose (BC) is a natural biomaterial synthesized by bacteria. It possesses a unique structure of cellulose nanofiber-weaved three-dimensional reticulated network that endows it excellent mechanical properties, high water holding capability and outstanding suspension stability. It is also characterized with high purity, high degree of crystallinity, great biocompatibility and biodegradability. Due to these advantages, BC has gained great attentions in both academic and industrial areas. This critical review summarizes the up-to-date development of BC production and application from an industrial perspective. Firstly, a fundamental knowledge of BC's biosynthesis, structure and properties is described, and then recent developments in the industrial fermentation of BC are introduced. Subsequently, the latest commercial applications of BC in the areas of food, personal care, household chemicals, biomedicine, textile, composite resin are summarized. Finally, a brief discussion of future development of BC industry is presented at the end.

## Introduction

**Figure 1:**
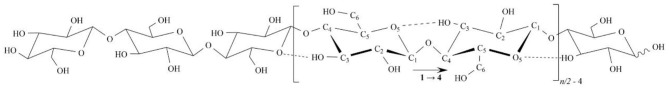
Chemical structure of cellulose molecule (*n* = degree of polymerization).

Cellulose is the most abundant natural polymers on the earth. It is chemically a linear homo-polysaccharide composed of repeating β-D-glucopyranose units linked by β-1,4 glycosidic bonds (Figure 1; Moon et al., 2011). Cellulose is sustainably synthesized by diverse life entities among the Plantae, Animalia, Fungi, and Bacteria Kingdoms (Iguchi et al., 2000; Abeer et al., 2014; Thomas et al., 2018). The key chemical profile of cellulose molecules is their plentiful hydroxyl groups existed along the polymer chain (one anhydroglucose has three hydroxyl groups; Ross et al., 1991). As a result, a large number of hydrogen bonds are formed between hydroxyl groups and oxygen atoms of anhydroglucose units (Pogorelova et al., 2020). The hydrogen bonding and van der Waals force promote a parallel stacking of cellulose molecules into crystalline nanofibers, which further assemble into cellulose microfibrils (Chen W. S. et al., 2018). The supermolecular structure having hierarchical order imparts cellulose microfibrils excellent mechanical strength, allowing them to act as a reinforcing component strengthening the natural architectures of organisms (Klemm et al., 2018). In human history, cellulose-based materials obtained from wood, cotton, and bamboo have been widely used for thousands of years as fundamental engineering materials for paper, construction, energy, textile, and furniture. However, the inefficient use of cellulose no longer meets the requirement of human society development. Additionally, the tremendous use of non-degradable petroleum-based plastics has caused a world-wide serious problem of white pollution (Arena et al., 2011). Thus, a public appeal from individuals and governments for the use of renewable and degradable materials instead of petroleum-based ones is continuously growing. Therefore, the exploitation of more advanced and wide utilization of cellulose-based materials is urgently required.

In the recent decades, nanotechnology has gained a great progress regarding cellulose. Cellulose nanoparticles including cellulose nanocrystals and cellulose nanofibers are successfully extracted from the woods, cottons, and other plants by using chemical, mechanical, and/or enzymatic methods (Klemm et al., 2011). Since the petroleum-based polymers are non-renewable and degradable to cause energy constraint and white pollution, cellulose nanoparticles are an ideal material based on which to build up a new biopolymer composites industry (Mokhena and John, 2020). Cellulose nanoparticles have a larger mechanical strength but a lower mass density compared with steel (Abitbol et al., 2016). They also possess an extremely low coefficient of thermal expansion similar with that of quartz (Hori and Wada, 2005). Moreover, they are also biocompatible and biodegradable (Roman, 2015). These advantages make cellulose nanoparticles become an attractive nanomaterial in both academic and industrial areas. To date, several kinds of cellulose nanofibers like 2,2,6,6-tetrametylpiperidine-1-oxyl (TEMPO)-oxidized (Isogai et al., 2011), carboxymethylated (Im et al., 2019), and phosphorylated cellulose nanofibers (Noguchi et al., 2020) have been developed to the stages of commercial production and application.

Beyond cellulose nanoparticles obtained from plants, algae, fungi, and bacteria can also produce cellulose, namely microbial cellulose (Abeer et al., 2014). Among them, bacterial cellulose (BC) is a natural nanomaterial produced by some species of bacteria (Reiniati et al., 2017). In comparison, the plant cellulose nanoparticles are obtained via the top-down methods, meanwhile BC is bottom-up synthesized nanofibers. To date, BC has been successfully produced via industrial fermentation (Keshk, 2014). BC is chemically equivalent to plant cellulose, but it has high degree of crystallinity and high purity (free of lignin, hemicellulose, pectin, and other biogenic components) as well as a unique structure of cellulose nanofiber-weaved three-dimensional (3D) reticulated network (Jozala et al., 2016). The unique structure endows BC distinct properties involving high wet tensile strength, large surface area, high water holding capacity, excellent permeability, flexibility, elasticity, and durability (Romling and Galperin, 2015). These advantages make BC an ideal candidate for renewable sources of cellulose materials. Indeed, BC has been commercially produced and used in our life.

This critical review introduces the up-to-date development of BC from an industrial perspective. The biosynthesis, structure and properties of BC are firstly described, and then the development of industrial production of BC is presented. Finally, the commercial applications of BC in the areas of food, personal care, household chemicals, biomedicine, textile, and composite resin are summarized.

## Biosynthesis and Assembly of BC

BC can be synthesized by a series of bacteria such as the genera *Gluconacetobacter, Aerobacter, Rhizobium, Sarcina, Azotobacter, Agrobacterium, Pseudomonas*, and *Alcaligenes* (Jonas and Farah, 1998). Among them, *Gluconacetobacter xylinu*s (*G. xylinus*, previously named *Acetobacter xylinus*) is the earliest discovered and most widely studied microorganism to produce BC (Zhong et al., 2013). It is a Gram-negative, aerobic and rod-like bacterium discovered by Brown (1886). In the latest bacterial systematics, *G. xylinus* has been combined into the genus *Komagataeibacter*, which is named in honor of Dr. Kazuo Komagata for his contribution to the bacterial systematics of acetic acid bacteria (Yamada et al., 2012). Therefore, *G. xylinus* is also called as *Komagataeibacter xylinus* (*K. xylinus*) now. The efficiency of *G. xylinus* to produce BC is extremely high. A single bacterium *G. xylinus* enables to polymerize 200000 glucose molecules into β-1,4 glucan chains and meanwhile arrange the polymer chains into nanofibers in 1 s (Chen et al., 2011). Due to the high yield, *G. xylinus* has been employed as a model microorganism for the mechanism study of BC synthesis and as industrial strains for the commercial fermentation (Keshk, 2014). Interestingly, a cell-free enzyme system is also developed to produce BC, which might transform into a cell-free factory for BC production in the future. The cell-free enzyme system is developed from BC-producing strains, and contains the whole enzymes and cofactors required for BC synthesis. The quantitative analysis reveals that the system produces BC with a higher yield than the corresponding bacteria (Ullah et al., 2015). Further study demonstrates that the cell-free enzyme system produces BC via an anaerobic biosynthesis process, and the premature BC pellicles formed in the culture media move to the air-liquid interface and assemble into a sheet (Kim et al., 2019).

**Figure 2:**
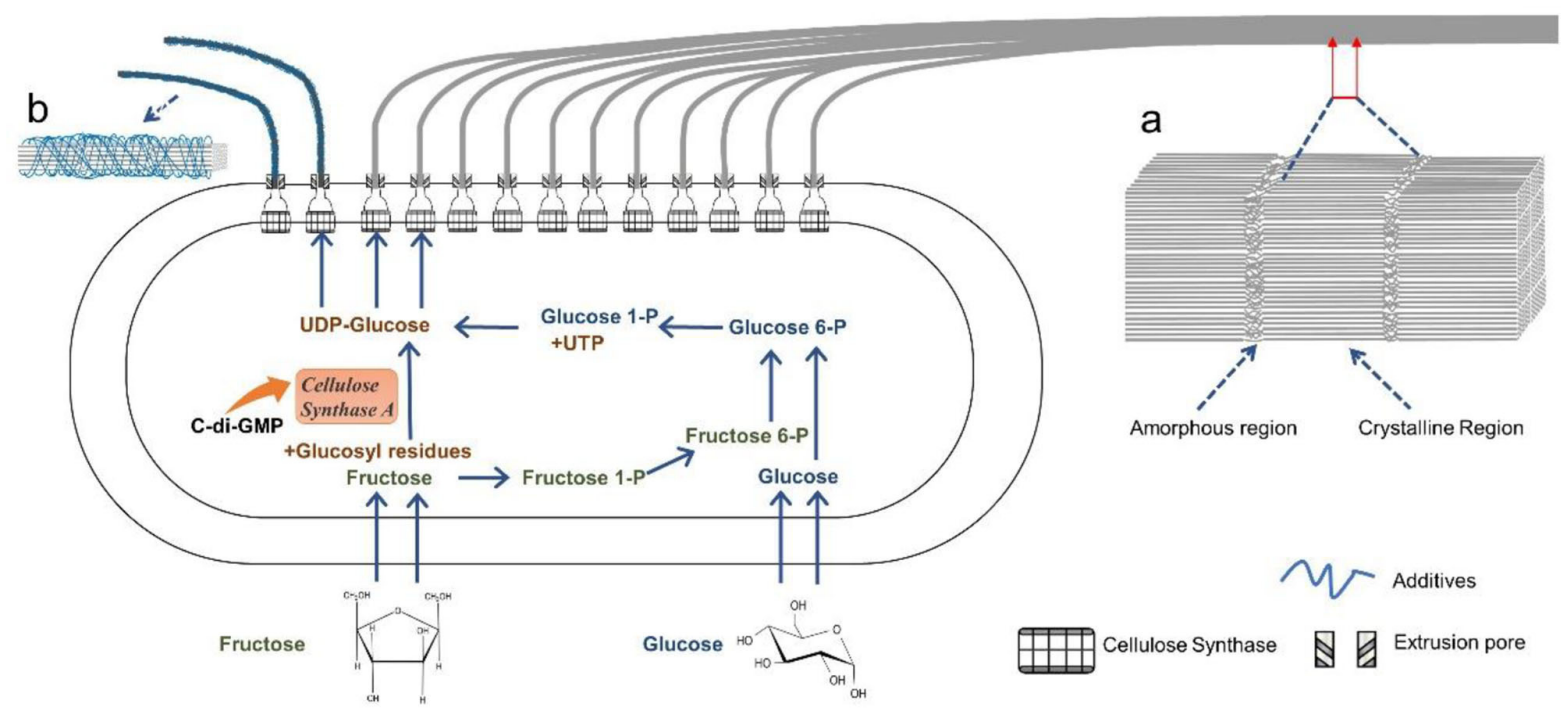
Schematic illustration of the intracellular biosynthesis of cellulose molecules and the extracellular assembly of cellulose molecules into nanofibers by a bacterium. A ribbon-like nanofiber is produced in standard fermentation **(a)**, and loosely-gathered nanofibers are harvested in the presence of additives **(b)**.

**Figure 3:**
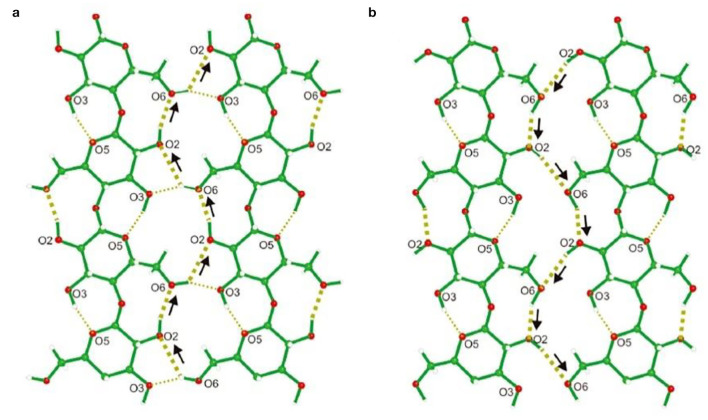
Hydrogen bonding network A **(a)** and B **(b)**. Thick dotted lines show the hydrogen bonding cooperation, and thin dotted lines indicate intra- and inter-molecular hydrogen bonds. The arrows highlight the donor-acceptor-donor directions. Reproduced with permission from Nishiyama et al. (2008).

However, the biosynthesis of BC by *G. xylinus* is complicated. It can be divided into two stages: (I) the intracellular polymerization of glucose molecules into cellulose polymers, and (II) the self-assembly of cellulose polymer chains into crystalline nanofibers (Figure 2; Czaja et al., 2007). The biochemical synthesis of cellulose occurs in bacteria, which commonly contains four enzyme catalyzed reactions (Figure 2; Moniri et al., 2017; Portela et al., 2019): (a) a glucose molecule is first converted to glucose 6-phosphate (glucose 6-P) by glucokinase; (b) a glucose 6-P molecule is isomerized into glucose 1-phosphate (glucose 1-P) by phosphoglucomutase; (c) a glucose 1-P molecule reacts with uridine triphosphate (UTP) to generate uridine diphosphate glucose (UDP-glucose), which is catalyzed by pyrophosphorylase; (d) UDP-glucoses are finally polymerized into linear β-1,4 glucan chains, which is catalyzed by cellulose synthase. If other types of carbon sources such as disaccharides are used for BC production, they are first hydrolyzed into monosaccharides such as glucose and fructose (Mohammadkazemi et al., 2015; Singhsa et al., 2018). If fructose used as carbon source, it is converted into fructose1-phosphate (fructose1-P), fructose-6-phosphate (fructose 6-P), and glucose 6-P via a number of enzymatic reactions (Figure 2; Chao et al., 2001). After the intracellular polymerization, the cellulose polymer chains are ejected out of the cell membrane by bacteria, following by a self-assembly process driving by van der Waals force and the intra- and inter-molecular hydrogen bonding between hydroxyl groups and oxygen atoms in the anhydroglucose units (Figure 3; Nishiyama et al., 2008; Ruan et al., 2016). The bacterium *G. xylinus*, likes a missile submarine, holds 50–80 terminal complexes (TGs) aligned along the long axis of bacterial cell (Figure 2; Kimura et al., 2001; Krasteva et al., 2017). From the TGs, the cellulose polymer chains are sprayed out, and then self-assemble into semi-crystalline nanofibers (Figure 2). In detail, the process involves two steps: (a) the cellulose molecules extruded from the same extrusion pore first assemble into a single elementary nanofiber with a diameter size of ~1.5 nm; (b) the elementary nanofibers are then gathered into a ribbon-like nanofiber with 3–4 nm thickness and 70–80 nm wideness in the cross-section (Figure 2; Portela et al., 2019). The ribbon-like microfibers further weave into 3D reticulated network to generate a gelatinous pellicle floating at the surface of culture media in a static fermentation, which innately provides an oxygen-rich, humid, and safe environment for the non-motile and aerobic bacteria to grow well and protects them from drying, radiation, and damage (Portela et al., 2019).

## Structure and Properties of BC

**Figure 4:**
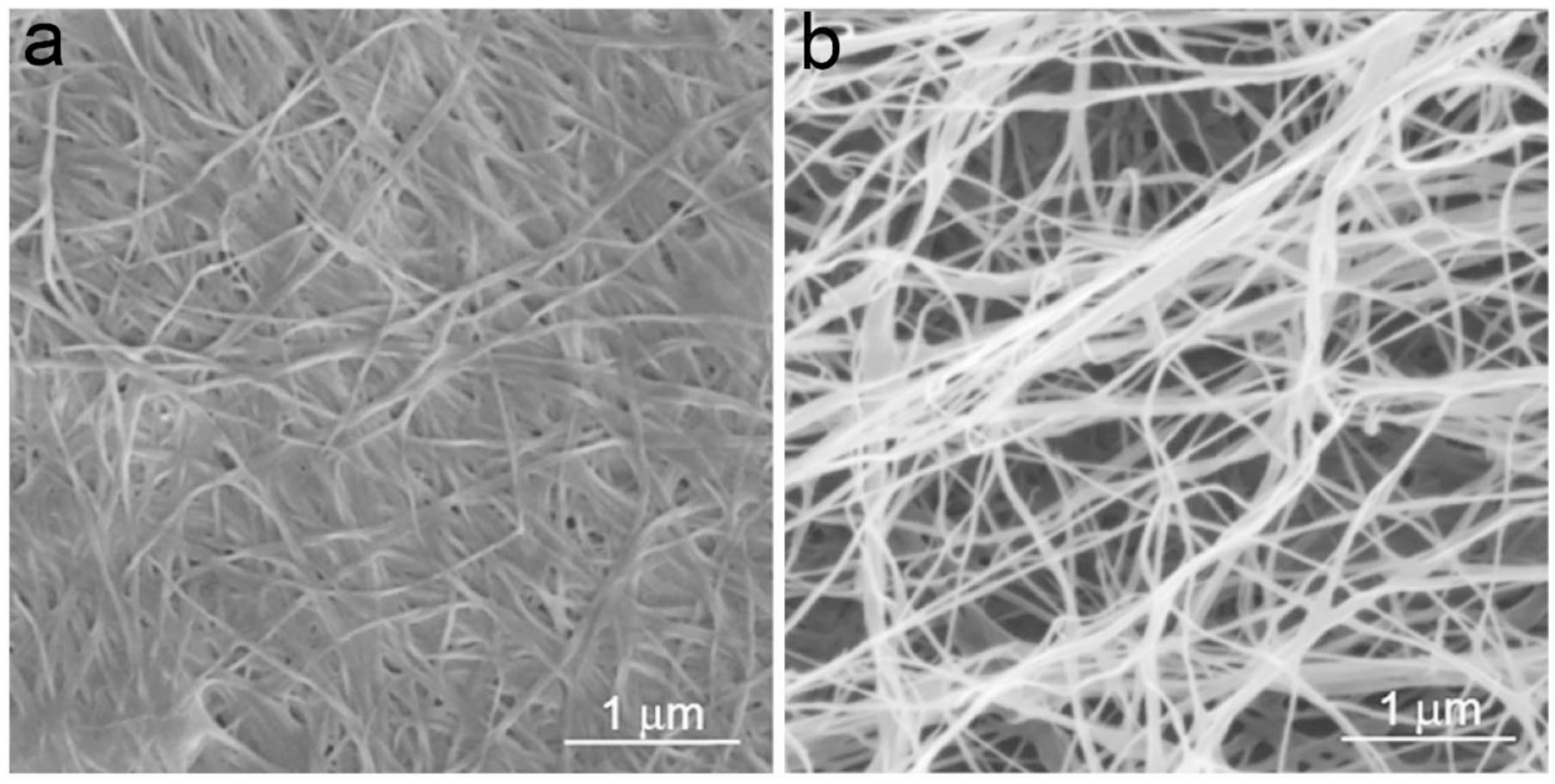
SEM images of BC. **(a)** BC pellicle harvested from static fermentation and **(b)** nanofibers detached from BC pellicle obtained via sonication. Reproduced with permission from Ruka et al. (2014).

**Figure 5:**
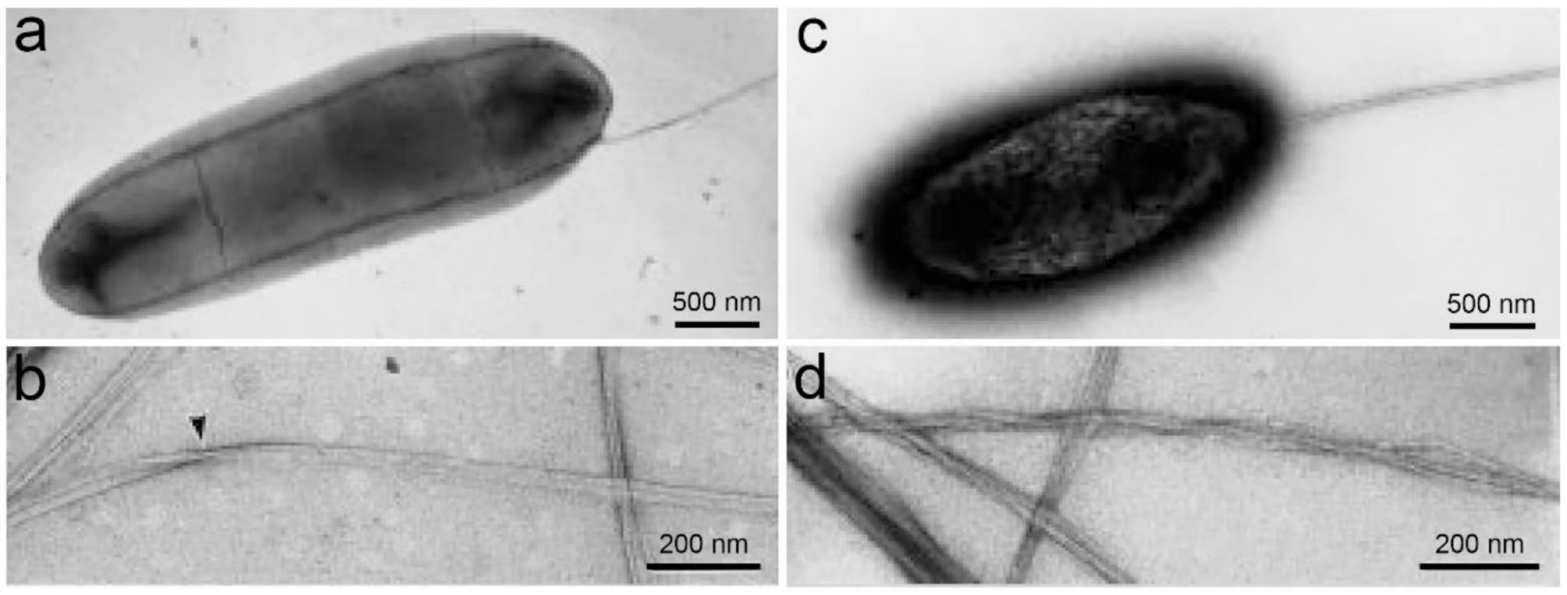
BC structure influenced by acetyl glucomannan. **(a,b)** A negatively-stained bacterium *G. xylinus* producing BC in HS medium **(a)**, and the produced BC nanofibers having a ribbon-like structure with striations and twists **(b)**. **(c,d)** A negatively-stained bacterium *G. xylinus* producing BC in HS medium containing acetyl glucomannan **(c)**, and the produced BC is a bundle of loosely-gathered nanofibers. Reproduced with permission from Tokoh et al. (1998).

Structurally, BC is ribbon-like cellulose nanofibers that further weave into 3D reticulated network (Figure 4; Ruka et al., 2014). It is characterized with high purity, high degree of polymerization and high crystallinity (Choi and Shin, 2020). BC is free of the symbiotic components in plants such as lignin, hemicellulose, and pectin. The raw pellicle harvested from microbial fermentation contains bacterial cells, residual nutrients and metabolic by-products, which can be easily detached from BC network to yield highly pure product. BC generally has a higher degree of polymerization than plant cellulose (Tabuchi et al., 1998). It varies depending on a series of factors including bacterial genera/strains, fermentation conditions, and nutrient sources (Tahara et al., 1997). BC also has a high crystallinity up to 90% (Sijabat et al., 2020). Like plant cellulose, there are also both crystalline and non-crystalline regions in the structure of BC (Moon et al., 2011). Since BC has high crystallinity, the crystalline regions are the major component of BC structure with short disordered sections as intervals, which probably attributes to both the high mechanical strength and flexibility of BC. The crystalline cellulose has different polymorphs including cellulose I, II, III, and IV (O'Sullivan, 1997). Among them, cellulose I exists in the natural products (Moon et al., 2011). Cellulose I also has two kinds of crystal structures, triclinic (Iα) and monoclinic structure (Iβ) (Azizi Samir et al., 2005). It is demonstrated that cellulose Iα can be irreversibly converted into cellulose Iβ in an alkaline solution by hydrothermal treatment, which suggests that cellulose Iβ represents a relative lower thermodynamic stability than cellulose Iα (Watanabe et al., 2007; Kose et al., 2011). Moreover, the hydrogen binding in cellulose Iα and Iβ is different, which should attribute to the differences in their thermodynamic stability (Moon et al., 2011). In natural products, the two allomorphs co-exist at different ratio proportions in biological species (VanderHart and Atalla, 1984). For instance, cellulose Iα is rich in BC with a mass fraction of ~0.6, while cellulose Iβ is dominant in the higher plants with a mass fraction of ~0.8 (Drahushuk et al., 1997). In bacteria, the proportions of cellulose Iα also vary depending on the genera and strains (Drahushuk et al., 1997). Moreover, the factors in fermentation such as temperatures, stirring and additives also influence the ratio proportions of cellulose Iα and Iβ in BC (Yamamoto and Horn, 1994; Kose et al., 2011). For instance, additives such as carboxymethylcellulose (CMC), xyloglucan, and acetyl glucomannan can disturb the crystallization and assembly of BC, resulting in the changes of both component and morphology (Tokoh et al., 1998; Chen et al., 2011). Moreover, glucose derivatives can also be integrated into BC during the fermentation (Gao et al., 2019). As a result, thinner nanofibers majorly composed of cellulose Iβ and coated with the additives are obtained (Figure 2; Yamamoto et al., 1996). Tokoh et al. have done a detail study over the structure and morphology changes of BC in the presence of acetyl glucomannan (Tokoh et al., 1998). A single bacterium *G. xylinus* produces a tightly-assembled nanofiber in the Hestrin-Schramm (HS) medium, but it sprays out loosely-gathered nanofibers in HS medium containing acetyl glucomannan (Figures 5a,b; Tokoh et al., 1998). The zooming-in images reveal that BC obtained from HS medium is an orderly-assembled and ribbon-like nanofiber with striations and twists in the structure, and BC harvested from acetyl glucomannan-containing medium is a bundle of loosely-gathered nanofibers with obvious gaps among them (Figures 5c,d; Tokoh et al., 1998). Ul-Islam et al. have done a comparative study over the properties of regenerated BC and plant cellulose (Ul-Islam et al., 2019). They demonstrate that the regenerated BC gel shows a better porosity, water absorption capability and water retention ability than that of plant cellulose. Moreover, the mechanical, thermal, and physiological properties of the regenerated BC gel are also better than these properties of the regenerated gel of plant cellulose.

BC is characterized with the unique structure of nanofiber-weaved 3D reticulated network (Yamanaka and Sugiyama, 2000). The reticulated network originally provides a safe and nutrient- and oxygen-rich living environment for bacteria (Portela et al., 2019). In the term of performances, the unique structure endows BC outstanding mechanical properties, high water holding capability, high suspension stability, and excellent gas permeability (Liu et al., 2020). It is demonstrated that BC has a very high wet tensile strength, although it varies depending on the genera and strains of bacteria as well as the fermentation conditions (Krystynowicz et al., 2002; Yuan et al., 2018). Recently, the mechanical properties of wet BC pellicles and dry films are well-determined by Natalia et al. (Pogorelova et al., 2020). The wet BC pellicles have an average Young's modulus of 1.5 ± 0.3 MPa and an average elongation of 49.0 ± 3.6% (Pogorelova et al., 2020). The dry BC films show a higher Young's modulus of 428.0 ± 24.1 MPa but a decreased deformability (7.5 ± 0.4%; Pogorelova et al., 2020). After rehydration, the BC films represent a compromise Young's modulus of 250.4 ± 20.1 MPa and an elongation of 18.5 ± 0.8% (Pogorelova et al., 2020). The water holding capability of BC wet films ranges from 60 to 700 times of its dry weights (Portela et al., 2019). In general, BC pellicles obtained from static fermentation is composed of ~1 wt % BC and ~99 wt % water (Yamanaka et al., 1989). Such high-water holding capability should attribute to the plentiful hydroxyl groups of cellulose. It is suggested that only 10% of the water molecules in BC pellicles are free, and the others are trapped by cellulose hydroxyl groups via hydrogen bonding (Mariani et al., 2007). Moreover, it is indicated that water in BC pellicle seems to be trapped in small “lakes” rather than form continuous phase throughout the pellicle (Mariani et al., 2007). BC produced from agitated fermentation is generally a pot of millimeter-scale small pellets with irregular shapes (Gorgieva, 2020). Such small pellets enable to suspend diverse particulates without obviously enhancing the viscosity, and moreover they exhibit excellent suspension stability to tolerate acid, salts, and ethanol (San-Ei Gen, 2020). In the wet condition, BC is also demonstrated to permit water vapor and gas exchange but do not allow liquid to transport, which also benefits a series of applications especially in biomedical areas (Gorgieva and Trcek, 2019; Pang et al., 2020). BC is naturally non-toxic and biocompatible (Roman, 2015). The 3D reticulated network of BC is similar with the collagen fiber-weaved extracellular matrix, which is also beneficial to its biocompatibility (Torres et al., 2012). BC represents good hemocompatibility (Andrade et al., 2011). The *in vivo* studies demonstrated that BC has no foreign body reaction and no chronic inflammatory during long-term implantation (Torres et al., 2012). The above-mentioned properties along with the other advantages such as flexibility, elasticity, durability, and biodegradability make BC a unique and universal nanomaterial in diverse applications.

## Production and Purification of BC

**Figure 6:**
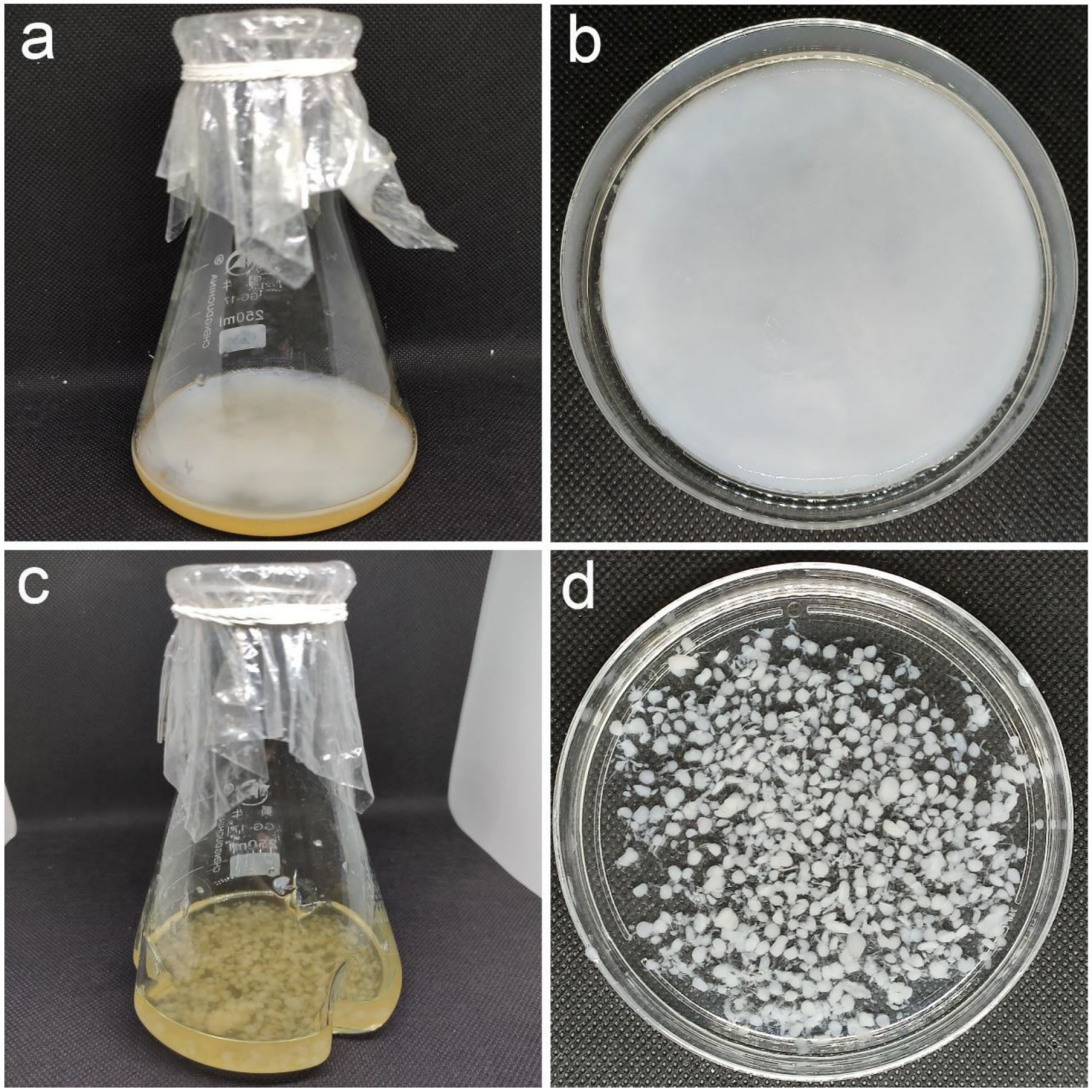
BC produced via static and agitated fermentation. **(a,b)** BC pellicle formed at the air-liquid interface of the medium in a static fermentation **(a)** and the purified BC pellicle with uniform texture **(b)**. **(c,d)** BC pellets fully filled in the medium in an agitated fermentation **(c)** and the purified BC pellets with irregular shapes.

BC is majorly produced by two kinds of methods, static and agitated fermentation (Figure 6). The choice between the methods for BC production depends on the application scenarios as the morphologies and properties of BC yielded by the two methods are very different (Islam et al., 2017; Pang et al., 2020). In a static fermentation, a gelatinous pellicle is formed at the air-liquid interface of the culture media (Figures 6a,b). In an agitated fermentation, small irregular pellets are fully suspended in the culture media (Figures 6c,d). The bacterial strains cultured in static fermentation represent a higher genetic stability to continuously produce BC in high yield, but the production efficiency is limited by fermentation method (Ross et al., 1991). The agitated fermentation is easily amplified to a large scale of industrial production (Chen G. et al., 2018), but it frequently induces an adverse conversion of bacteria into the non-cellulose producing mutants that reduce the yield (Sani and Dahman, 2010). Although BC gels produced by the two methods show very different macroscopic morphologies, they maintain the same microstructure of 3D reticulated network (CPKelco Inc, 2020). Furthermore, BC obtained in agitated culture has a lower degree of polymerization and a lower crystallinity in comparison with that produced in static culture (Watanabe et al., 1998). The CP/MAS ^13^C NMR analysis reveals that the ratio proportion of cellulose Iα in BC obtained in agitated culture is lower than that in BC produced in static culture, and accordingly the amount of cellulose Iβ in BC obtained in agitated culture is increased (Watanabe et al., 1998). The mechanical properties of BC produced by the two methods are also different. BC yielded in static culture represents a higher Yong's modulus compared with that produced in agitated culture (Krystynowicz et al., 2002). However, BC from agitated culture has a higher water holding capability and suspension viscosity than that from static culture (Krystynowicz et al., 2002). In view of the differences in morphologies and properties, the applications of BC produced by the two methods are also dissimilar. For instance, the static fermentation is preferred for the production of raw materials requiring fixed geometries, well wet tensile strength and high-water holding capability, such as nano de coco, wound dressing, and face mask etc. On the other hand, BC produced by agitated fermentation represents the superiority in suspending stability, which is majorly used for particulate suspension in the beverages.

For both static and agitated methods, culture medium is the most important part for BC production, which not only provides the necessary nutrients for bacterial growth and BC production, but also significantly impacts structures and yields of BC as well as its mechanical and physical properties (Jozala et al., 2016). A typical fermentation medium is at least comprised of a carbon source, a nitrogen source, and certain nutrient elements such as phosphorus, potassium, sulfur, and magnesium (Andriani et al., 2020). The typical culture medium used for BC production is first described by Hestrin and Schramm (1954). It contains 2.0 wt % glucose, 0.5 wt % peptone, 0.5 wt % yeast extract, 0.27 wt % Na_2_HPO_4_, and 0.115 wt % citric acid (Hestrin and Schramm, 1954), in which glucose serves as a carbon source, peptone, and yeast extract act as nitrogen sources. The pH value of the medium is adjusted to 6.0 by using HCl or NaOH.

However, the production cost is too high to impede the industrial process of BC. There are two routes to reduce the production cost. One way is to enhance the BC production efficiency, and the other way is to seek cost-effective nutrient sources as the culture media account for ~30% of the total production cost (Rivas et al., 2004). To date, there are several ways to promote the yield of BC: (I) isolating new bacterial strains that efficiently produce BC from the nature (Yang et al., 2013; Aydin and Aksoy, 2014); (II) employing traditional mutagenic methods (such as ultra-violet and chemical mutagenesis) and genetic engineering techniques to screen high-yield strains (Li et al., 2016); (III) optimizing the culture conditions (Krystynowicz et al., 2002; Cheng et al., 2009). The standard HS medium is expensive due to the high-cost of nutrients. However, the bacteria can be fed with different carbon sources (such as glucose, sucrose, fructose, mannitol, arabitol, and molasses) and nitrogen sources (such as yeast extract, peptone, and corn steep liquor; Keshk and Sameshima, 2005; Buldum et al., 2018). In recent years, the agricultural and/or industrial residues such as coconut water/milk, beet molasses, waste beer yeast, rotten fruit culture, liquid fermentation wastewater, and citrus juice have also been successfully exploited as nutrient sources for BC production (Kongruang, 2008; Wu and Liu, 2013; Velásquez-Riaño and Bojacá, 2017; Cao et al., 2018; Julia et al., 2019). The use of these substituted nutrients significantly reduces the production cost of BC and additionally alleviates environmental pollution induced by the improper discard of the industrial wastes.

After fermentation, the raw BC pellets are not pure, which contain bacterial cells, nutrient residues, and metabolic substances. Thus, a purification process is required to obtain high purity of BC. Cellulose in plant cells tightly co-exists with hemicellulose, lignin and pectin, which are not easily removed. However, BC can be easily purified via a routine method (Moniri et al., 2017). It involves three steps: (I) treat BC pellets with alkaline solutions at 100°C for 15–20 min to remove bacterial cells; (II) isolate BC pellets from the alkaline solution; (III) wash BC pellets by distilled water to recover neutral pH value. The endotoxin in BC can also be controlled under 20 endotoxin units/device, which is acceptable as specified by the US Food and Drug Administration (FDA; Petersen and Gatenholm, 2011).

## Industrial Production of BC

Currently, BC has been industrially produced and widely used in diverse areas. Donini et al. estimate that the fermentation production of BC can achieve a comparable production efficiency with the growth of plant cellulose when the yield of BC reaches up to 15 g/L in 50 h (Donini et al., 2010). Moreover, the production area required for BC fermentation is much smaller than that needed for the plant growth, and the purification of BC is simple and less of pollution compared with the procedure of cellulose extraction from woods. Additionally, the agricultural and industrial wastes are commonly utilized in the commercial fermentation, which not only reduces the cost but also diminishes the waste-induced pollution (Hussain et al., 2019). Therefore, BC can be a competitive alternative for plant-based cellulose nanofibers in certain application areas.

BC-based products have gained a huge market success, especially in food areas. According to a report from ResearchMoz, the BC market is around US$ 207.36 million in 2016, and is expected to be US$ 497.76 million in 2022 and to surpass US$ 700 million in 2026 (ResearchMoz and QYResearch, 2017). Nata de coco is so far the main commercial product of BC, which is harvested from static fermentation by using coconut water as nutrient source. It is sold in the terms of slabs and diced pieces in the market depending on the customer's requirements. The price of nata de coco is varied in a range of US$ 200–1000 per ton, which is changed among different manufacturers and is dependent on the forms and quality of final products (Ul-Islam et al., 2020). A techno-economic analysis of industrial-scale fermentation of BC has been performed using Super-Pro Designer software by Dourado et al. (2016a). The software estimated that the capital investment of an industrial manufactory to produce 504 tons of BC per year is around US$ 13 million. The manufacturing cost of BC is estimated to be US$ 7.4 million per year, and the net profit to be US$ 3.3 million per year. Although BC production is highly capital-intensive, researchers, and manufacturers have been working on the development of new ways to reduce the production cost via isolation of high yield of strains, optimization of fermentation reactors, and utilization of low-cost nutrient substrates (Ul-Islam et al., 2020).

**Figure 7:**
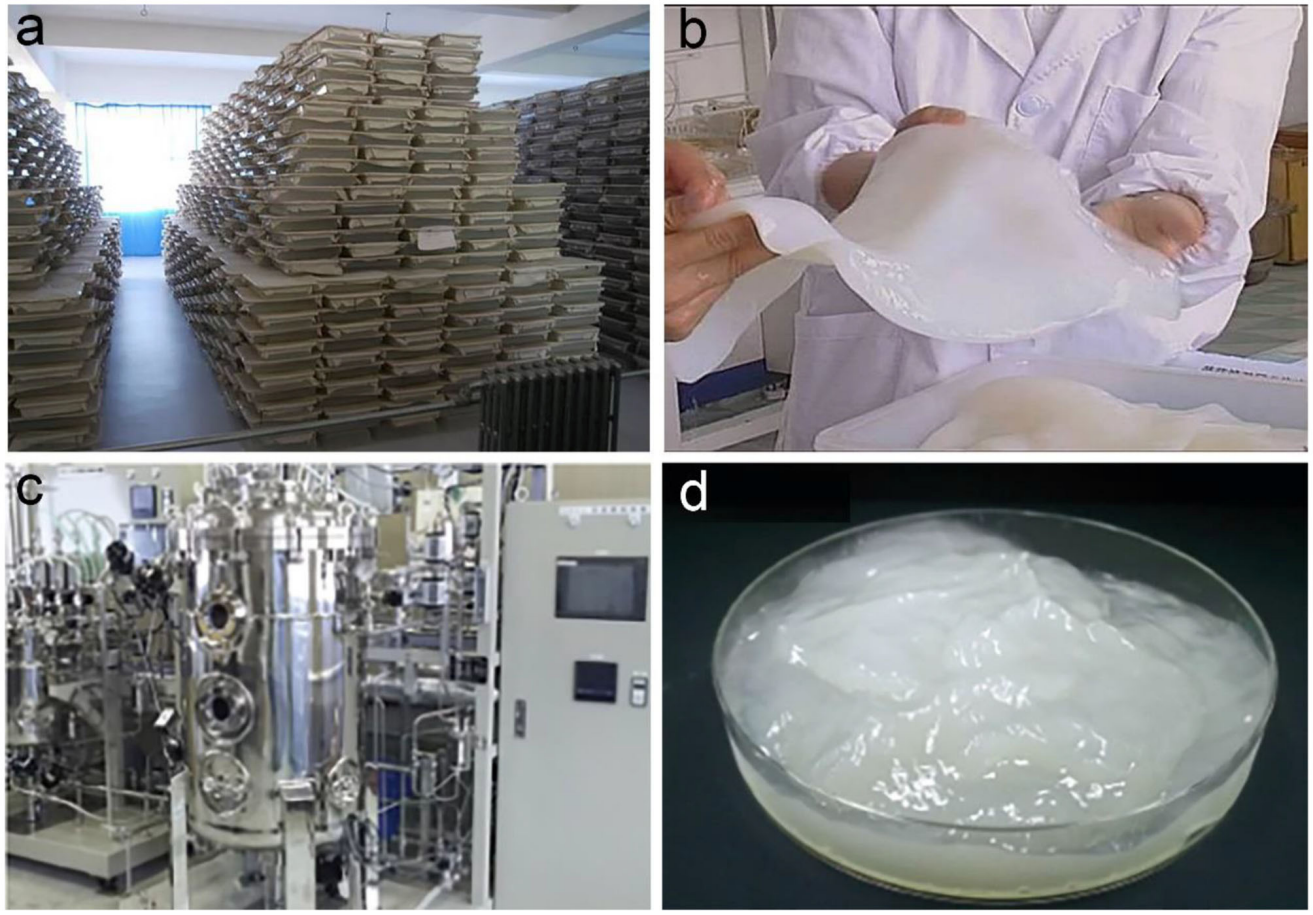
Industrial production of BC via static and agitated fermentation. **(a,b)** Shallow tray fermentation used for industrial production of BC **(a)** and the purified BC pellicle **(b)**. Reproduced with permission from Hainan Yeguo Foods Co., Ltd (2020). **(c,d)** Agitated fermentation used for industrial production of BC **(c)** and the harvested BC slurry **(d)**. Reproduced with permission from Kusano Sakko Inc (2020).

To date, both the static and agitated fermentations have been successfully used for the industrial production of BC (Figure 7). The *G. xylinus* strains are employed as the main bacteria for BC production (Keshk, 2014). To reduce the cost of culture media, the food industrial wastes such as coconut water/milk and beet molasses are used as the nutrient sources (Zhong, 1996; Kusano Sakko Inc, 2020). The static fermentation is first used for the industrial production of BC, which can be early traced back to 1970s in Philippine to produce nata de coco (Iguchi et al., 2000). The nata de coco holds a series of unique properties of jelly-like morphology, cool and crisp tastes, and near-zero cholesterol (Ullah et al., 2016). Therefore, it becomes into a very popular raw food material that is widely used as dessert, additives to drinks, sauce and stuffing, and garnish to dishes (Azeredo et al., 2019). Later, the nata de coco is prevalent in Japan and other south Asian countries, which accelerates the industrial production of BC. In China, the industrial production of BC is initiated by Zhong (1996). She isolated a *G. xylinus* strain from the fermented coconut water, and then started up Hainan Yeguo Foods Co. Ltd. for production and application development of BC (Hainan Yeguo Foods Co., Ltd, 2020). The *G. xylinus* strain 323 is stored in China General Microbiological Culture Collection Center (No.1186; Zhong, 2009). They majorly adopt the static fermentation to produce BC (Figures 7a,b). Up to now, this company has developed into one of the biggest manufacturers for BC products in the world.

A series of problems are encountered during the industrialized process of static fermentation of BC. The optimum fermentation temperature of *G. xylinus* is around 30°C (Son et al., 2001), and thus the BC production is majorly restricted in the regions of Southeast Asia such as Philippines, Indonesia, Vietnam, and Thailand. In Hainan province of China, heating is required to maintain a high temperature for BC production in winter, which inevitably increases the energy consumption. Moreover, high temperature frequently induces microbial contamination, causing a series of issues related to food safety, production capacity, and environment pollution. To solve the problem, Zhong and co-workers utilize a low-temperature domestication method to reduce the optimum fermentation temperature of bacteria for BC production, and they successfully screen out a low-temperature resistant strain that can produce BC at 10–20°C with high yield (Zhong, 2009). This technology enables BC production to be carried out in the cold regions.

The agricultural and industrial wastes are used as nutrient sources for BC production to reduce the cost. So far, the major nutrient source for commercial production of BC has still been coconut water (Hainan Yeguo Foods Co., Ltd, 2020). Although the coconut water is once an industrial waste, the huge market demand makes it scarce, thus causing its price to rise. Zhong et al. develop a two-step fermentation method to use other raw materials instead of coconut water (Zhong, 2008b). They find the culture media previously fermented by acetic acid bacteria or lactic acid bacteria can obviously enhance the yield of BC. Therefore, they utilize the agricultural or industrial wastes such as corncob, alcohol waste liquor, pineapple peel, citrus juice, and apple juice to conduct a former fermentation and then used for BC production. This method achieves a comparable yield with the fermentation by using coconut water, thus broadening the nutrient sources for BC production.

The cost of transportation and storage of BC is very high due to its high-water holding content (~99 wt %; Yamanaka et al., 1989). In order to low the transport and storage cost, the water in BC is reduced to ~10 wt % through a two-step compression method followed by an organic acid dipping treatment (Zhong, 2008a). The strategy prevents the destruction of the network structure in BC, and also maintains its excellent rehydration capability (rehydration rate up to 95%). The method not only reduces the transport and storage cost of BC, but also endows BC diverse flavor as raw food materials.

The agitated fermentation has also been successfully used for industrial BC production (Figures 7c,d). It has been commercially exploited as a thickener and/or suspending agent especially for suspension of particles due to its unique structure with 3D reticulated network (Swazey, 2014). The raw BC after purification from agitated fermentation is full of water, which is not suitable for transport and storage. CPKelco, a global company to produce nature-based hydrocolloids, produces BC named as fermentation-derived cellulose via agitated fermentation (CPKelco Inc, 2020). Initially, a wet cake form of BC is commercially produced by CPKelco, which contains 10–20% solids and the other balance water (Swazey et al., 2013). Furthermore, dry powder forms are also exploited by adding different compound additives (Swazey, 2014). Currently, a product with the trade name of CELLULON^TM^ Cellulose Liquid is assessable in its website, which is developed as hydrocolloid for suspension of actives, decorative particles, or perfumed nanoparticles with minimal influence to the viscosity (CPKelco Inc, 2020). A Japanese company, San-Ei Gen F. F. I., Inc. also provides BC via agitated fermentation with a trade name of Sun Artist^@^, which is also exploited as suspending agent majorly in food areas (San-Ei Gen, 2020). Kusano Sakko Inc., another company in Japan, has also produced BC with a trade name of Fibnano (Figures 7c,d; Kusano Sakko Inc, 2020). They use molasses, a waste by-product in sugar industry instead of coconut water for BC fermentation, as they produce sugar by using sugar beet and sugar cane. Since 2012, they have put great effort in the production and application development of BC, and has given out various potential applications including medical care, food, personal care products, special paper, and resin filler (Kusano Sakko Inc, 2020).

## Applications of BC

BC has been widely used in the commercial areas of food industry, personal care products, house hold chemicals, biomedicine, textile, composite materials etc. The detail of the applications in each area is described in the following sections.

## Applications in Food Industry

**Figure 8:**
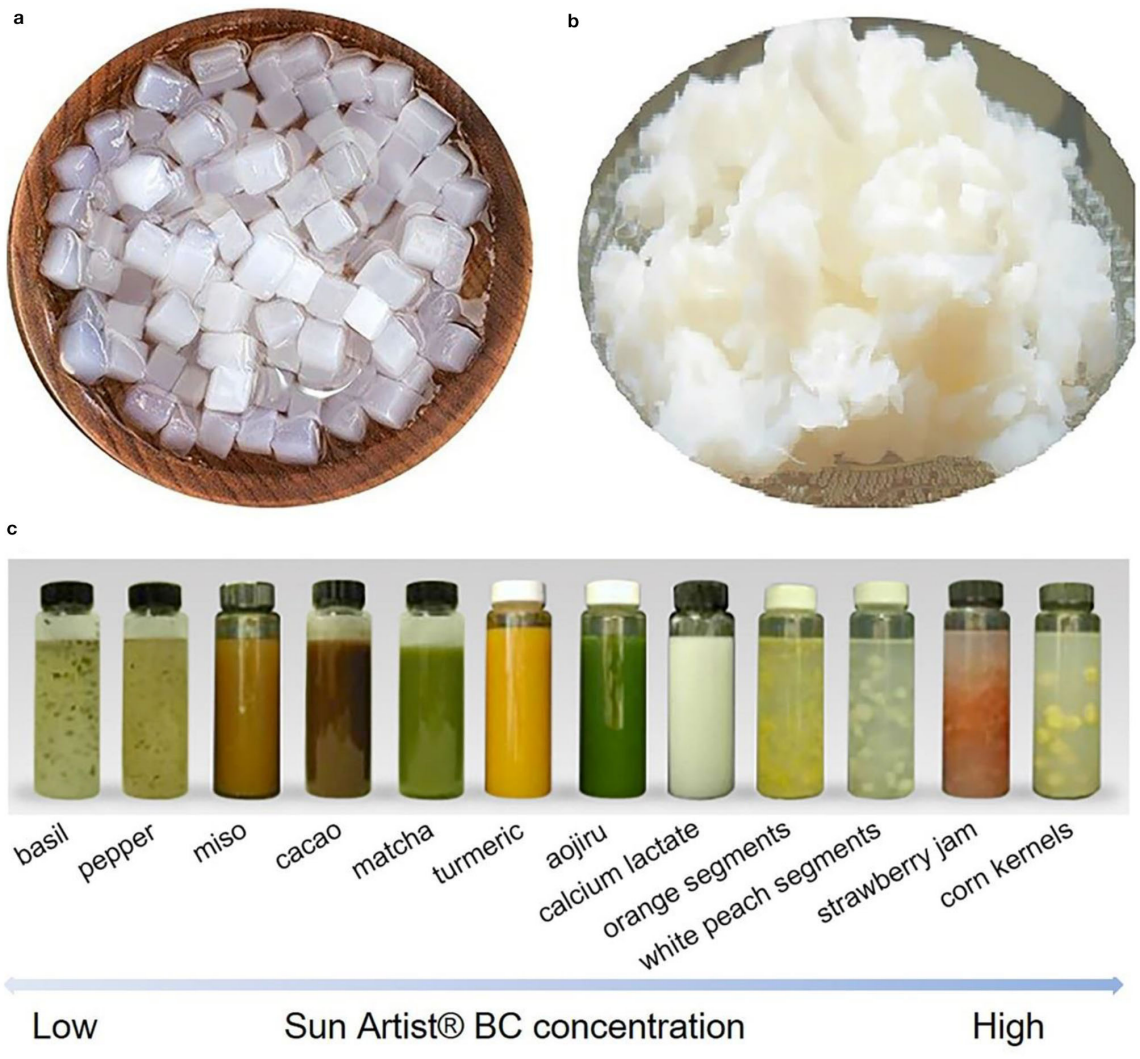
BC particles used in food products as additives. **(a)** Cube-shaped BC obtained from static fermentation followed by cutting process, and **(b)** the compressed ones harvested via a two-step compression method. Reproduced with permission from Hainan Yeguo Foods Co., Ltd (2020). **(c)** Sun Artist® BC used for the suspension of diverse solid food ingredients with high stability. Reproduced with permission from San-Ei Gen (2020).

BC has been considered as a “generally recognized as safe (GRAS)” food additives by FDA since 1992 (Shi et al., 2014). It has the potential uses in traditional dessert, low cholesterol diet, vegetarian meat, food/beverage additives, and food packaging etc. (Azeredo et al., 2019). In commercial applications, the forms and functions of BC are varied depending on the fermentation methods. BC produced from static fermentation with a jelly-like pellicle is majorly used as raw materials for food dessert and food ingredients (Figures 8A,B; Hainan Yeguo Foods Co., Ltd, 2020), and meanwhile BC obtained from agitated fermentation with hydrocolloid nature is exploited as thickeners and suspension agents in beverages (Figure 8C; San-Ei Gen, 2020). The human body lacks cellulase. Therefore, BC cannot be digested and absorbed in the gastrointestinal system, and is eliminated out of the body in the feces (Fontana et al., 2017).

The earliest application of BC in food can be dated to the 1960-1970s in Philippines (Iguchi et al., 2000). Nata de coco is praised for the high and pure fiber content, near-zero calories, and cholesterol counts (Ullah et al., 2016). Thus, it like the dietary fiber taken from daily foods to benefit the human health via reducing the risk of chronic diseases such as diabetes, obesity, and cardiovascular disease etc. (Anderson et al., 2009). Nata de coco is generally cut into cubes and pickled into different flavors (Figures 8A,B; Hainan Yeguo Foods Co., Ltd, 2020). Moreover, during the fermentation process, BC can be produced with different shapes and textures as well as diverse flavors by varying the fermentation conditions and/or the recipes of culture media, thus significantly enriching the variety of BC products (Gatenholm and Klemm, 2010). It is early enjoyed as a dessert due to the cool and smooth feeling, crunchy texture, as well as juicy taste. It is also exploited as an additive to endow the food new texture and flavor (Shi et al., 2014). Now, nata de coco can be found in a variety of foods such as drinks, yogurt, pies, sausages, and salads. It is early prevalent in Philippines, and then prevalent in Japan and other Asian countries in the 1990s (Dourado et al., 2016b).

On the other hand, BC is employed as a functional additive for the food industry. There are plenty of beverages and liquid foods that have particulate components such as milk beverages, coffee, porridge, and soybean milk, which need suspension. Generally, thickeners and surfactant agents like xanthan gum, pectin, CMC, soybean polysaccharide are added in the liquid products to suspend particulates (Dourado et al., 2016b). However, these formulations commonly represent a poor suspension stability, and are often troubled with transparency interferences and phase separation (John McArthur Swazey and Madison, 2010). Moreover, the high viscosity also causes unpleasant taste to customers. Therefore, new suspension agents with excellent dispersion stability and low viscosity are required. Under this condition, BC pellets produced by agitated fermentation are found to have the great characteristic to well fulfill the function to suspend particulates (Figure 8C; San-Ei Gen, 2020).

BC has a unique structure of nanofiber-weaved 3D reticulated network, which endows it excellent capability to suspend insoluble particulates with low viscosity (Swazey, 2014). It can well perform the suspension function at a low concentration, and it also works well even in the presence of high concentrations of surfactants and thickeners. Moreover, BC is suspended rather than solubilized in solution, and it is uncharged. This feature allows BC to be minimally affected by environment factors such as acidity and ionic strength (Swazey, 2014). Therefore, BC maintains the suspension capability in a wide range of pH values, and has great toleration with salts. Due to the high degree of crystallinity, BC also represents an excellent enzyme resistant (Torres et al., 2019). Finally, BC also exhibits undiminished suspension stability at a high temperature up to 80°C (San-Ei Gen, 2020). These advantages make BC a cost-effective and non-substitutable suspension agent for particulates.

To date, there are several companies such as CPKelco, San-Ei Gen, and Kusano Sakko have commercially applied BC from agitated fermentation as a suspension agent in the food areas. Originally, CPKelco commercially produces BC in the form of a wet cake typically with 10–20% solid and the other balance water (Swazey et al., 2013). In this formulation, sorbic acid is also added to prevent mold (Swazey et al., 2013). The wet cake is activated via high-speed shearing to recover the dispersed form in solution. Dry powder forms are also exploited by CPKelco with the trade name of AxCel®PX, AxCel®CG-PX, AxCel®PG, Cellulon^TM^PX, and “K”-named series (John McArthur Swazey and Madison, 2010). These dry powder forms are generally composed of BC and one or more surfactants and/or thickeners such as xanthan gum, CMC, pectin, carrageenan etc. (Swazey, 2014). These co-agents allow BC to recover the dispersed state when meet water. However, the co-agents somehow adversely affect the suspension stability of BC, as they are commonly charged polymers. When the dry powder used in a solution with low acidic value or high ionic strength, these charged polymers may become insoluble to lose their functions, and thus inversely cause the suspension system to become unstable or non-transparent (John McArthur Swazey and Madison, 2010). The patent (John McArthur Swazey and Madison, 2010) applied by CPKelco gives out a solution method that involves a polymer degrader within the dry powder form. The polymer degraders can be enzymes, oxidizers and metal ions, which can selectively degrade the co-agents without interfering the function of BC (John McArthur Swazey and Madison, 2010). San-Ei Gen has its BC product in a liquid form with the trade name of “Sun Artist®” (San-Ei Gen, 2020). San-Ei Gen well displays the functions and applications of the Sun Artist® BC in diverse beverages and liquid foods such as basil, pepper, miso, cacao, matcha, turmeric, aojiru, calcium lactate, orange segments, white peach segments, strawberry jam, and corn kernels (Figure 8C; San-Ei Gen, 2020). They have also demonstrated that BC is able to prevent the sedimentation of particulates for a long period (over 1 month), and BC has an excellent resistance to acid, salt and heat (San-Ei Gen, 2020). Kusano Sakko Inc. shows that they are trying to use BC as a thickener and emulsion stabilizers in the food areas. Taken together, these commercialized applications suggest that BC has a huge market in the food industry areas (Shi et al., 2014).

## Application in Personal Care Products and Household Chemicals

**Figure 9:**
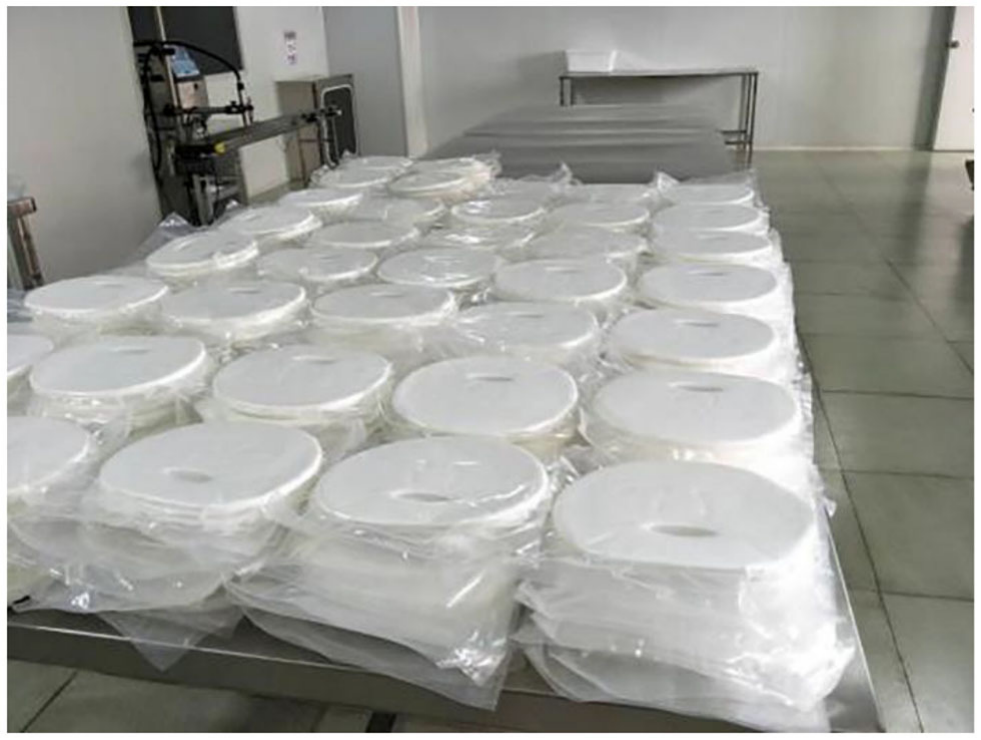
BC-based raw materials for face mask. Reproduced with permission from Hainan Yeguo Foods Co., Ltd (2020).

The second large application area of BC is personal care products and household chemicals (Bianchet et al., 2020). The component materials used in personal care products should be non-toxic and biocompatible. Thus, natural product with high purity and high safety is favored by costumers. BC is a natural product generated via microbial fermentation, which has been demonstrated to be highly biocompatible (Roman, 2015). In the personal care products, BC pellicles harvested from static fermentation have been exploited as raw materials for face masks (Figure 9; Hainan Yeguo Foods Co., Ltd, 2020). In comparison with non-woven cellulose or silk face masks, BC-composed face masks have more excellent water holding capability and give a favorable feeling of coolness and smoothness owing to its nanoscale 3D reticulated network (Amnuaikit et al., 2011; Pacheco et al., 2018). Moreover, the highly porous microstructure allows BC to load various nutrients and even ingredients with therapeutic functions (Chantereau et al., 2020). The porous microstructure also imparts BC pellicle a function to control the release of these entrapped agents (Numata et al., 2015; Perugini et al., 2018). Based on this function, the BC-composed face masks can also be used in cosmeceuticals and treatment of mild skin diseases (Almeida et al., 2014; Morais et al., 2019).

Moreover, BC obtained from agitated fermentation also serves as a suspension agent, like the function in foods, to offer an excellent suspension stability for particulates such as decorative micro beads, encapsulated fragrance, and encapsulated enzymes etc. (Swazey and Morrison, 2015a,b). Both companies of CPKelco and Kusano Sakko have exploited their products in the applications of liquid laundry, detergent, and personal care fields (CPKelco Inc, 2020; Kusano Sakko Inc, 2020).

## Application in Biomedical Areas

BC has great potential in diverse biomedical applications including wound dressing, artificial skin, dental implant, drug delivery, hemostatic materials, vascular grafts, scaffolds for tissue engineering, biosensor and diagnosis (Rajwade et al., 2015; Anton-Sales et al., 2019; Carvalho et al., 2019). The high purity and biocompatibility of BC are the prerequisite for all the biomedical applications. The endotoxin in BC is also well controlled under 20 endotoxin units/device, which meets the requirement of FDA for the *in vivo* usage (Petersen and Gatenholm, 2011). Moreover, BC possesses unique 3D reticulated network, which endows a series of advantages like large surface areas, excellent water holding capability, well liquid/gas permeability, remarkable mechanical properties and transparent nature (Thomas, 2008; Sulaeva et al., 2015). These distinctive characteristics make BC a very special material to demonstrate its superiority in biomedical applications. BC-based wound dressing devices have been successfully commercialized in the market, and there are also several products related to drug delivery, contact lens, vascular grafts, tympanic membrane replacement on the way of commercial transformation (Coelho et al., 2019).

Skin is the largest organ of the human body. It protects us from microorganisms, maintains body homeostasis, regulates body temperature, and feels sensations (Zhang et al., 2019). The diseased skin will lose these functions and cause severe consequences. There are diverse conditions to induce traumatic skin loss by either internal factors such as vascular disease, heart disease and diabetes, or external reasons such as accidents, suffering burn or scald and surgical operations (Vogelnest, 2017). To regenerate skin, a common treatment procedure involves a routine surgical treatment followed by complete coverage of the skin lesion by using wound dressing. An ideal wound dressing should maintain moisture of the wound lesion, eliminate exudates, allow perspiration and oxygen exchange, reduce electrolyte and protein loss, avoid infections, reduce pain, and finally accelerate wound healing (Portela et al., 2019). However, the conventional wound dressings such as gauze and synthetic materials can not satisfy these requirements.

**Figure 10:**
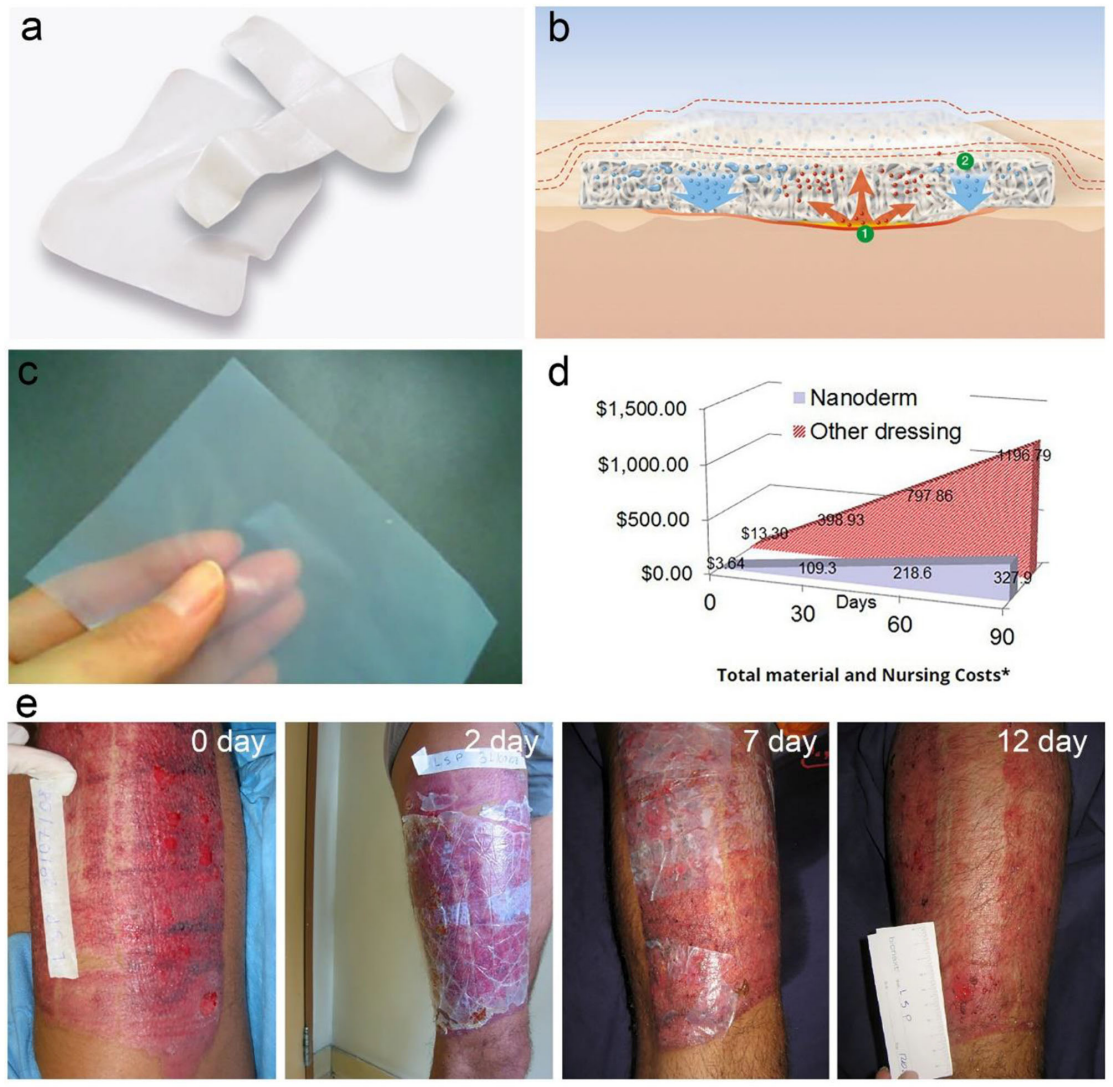
BC-based wound dressing. **(a)** Suprasorb® X wet wound dressing composed of 1.5–4.3% BC and the other balance water. **(b)** Scheme shows Suprasorb® X wound dressing enables to absorb exudate from the wound (①) and transfer moisture to other areas with little exudate (②). Reproduced with permission from Lohmann and Rauscher International GmbH & Co., 2020; Suprasorb® X Lohmann Rauscher International (2020), and Suprasorb® X + PHMB Lohmann Rauscher International (2020). **(c)** Nanoderm^TM^ dry wound dressing with flexible and semitransparent features. **(d)** Total material and nursing cost of Nanoderm^TM^ wound dressing vs. regular wound dressing. Reproduced with permission from Axcelon Dermacare Inc (2020). **(e)** Nanoderm^TM^ wound dressing applied to the skin donor site. The photographs from left to right are the initiated donor site, and 2, 7, and 12 days after treatment. Reproduced with permission from Axcelon Dermacare Inc (2020).

BC is initially exploited as wound dressing due to its well moisture control, high wet tensile strength, permeability, flexibility, semitransparent nature, and great biocompatibility (Curvello et al., 2019). After the practical use, it is found that BC has a series of additional advantages including eliminating exudates meanwhile allowing perspiration and gas exchange, reducing pain and loss of electrolyte and protein, preventing infections, and accelerating wound closure (Abeer et al., 2014). These distinguished advantages give birth to BC in the market of wound dressing devices. As a result, a series of BC-based wound dressings are commercialized under the trade-marks of Nanoderm^TM^, Bionext®, Membracell®, Suprasorb® X, Biofill®, Gengiflex®, Xcell® etc. (Abeer et al., 2014). BC-based wound dressings show higher efficiency in comparison with these traditionally-used gauze or synthetic materials, and are widely used for the treatment of arterial and venous ulcers, diabetic ulcers, pressure ulcers, burns, post-operative surgical wounds, skin grafts, skin graft sites, abrasions, lacerations etc. (Portela et al., 2019). BC-based wound dressings on the market are formulated in a moisture membrane or a dry film (Figure 10). For instance, the Suprasorb® X wound dressing is a wet pellicle composed of 1.5–4.3% of BC and the other balance water (Figure 10a; Lohmann and Rauscher International GmbH & Co., 2020; Suprasorb® X + PHMB Lohmann Rauscher International, 2020), which is commercialized by a German company, Lohmann & Rauscher International. The Suprasorb® X wound dressing enables to balance the moisture in the chronic wound lesion. It absorbs fluid exudate of 20–200% its weight from exuding wound lesions, and is able to transfer moisture to dry or necrotic wound lesions by more than 75% of its weight (Figure 10b; Lohmann and Rauscher International GmbH & Co., 2020; Suprasorb® X Lohmann Rauscher International, 2020). Suprasorb® X wound dressing is used for the treatment of non-infected superficial or deep wounds with low to moderate levels of exudate. The indications involve arterial and venous ulcers, diabetic ulcers, pressure ulcers, superficial 2nd degree burns, post-operative surgical wounds, skin grafts and skin graft sites, abrasions, lacerations (Lohmann and Rauscher International GmbH & Co., 2020; Suprasorb® X Lohmann Rauscher International, 2020). The benefits include reducing pain, exudate management, accelerated wound healing, high wearing comfort, better cosmetic outcome, and it is also cost-effective in use due to a long dressing change interval (Lohmann and Rauscher International GmbH & Co., 2020; Suprasorb® X Lohmann Rauscher International, 2020). Furthermore, they developed an advanced wound dressing containing polyhexamethylene biguanide (PHMB), a widely-used and safe antimicrobial agent for the care of microorganism-infected wound (Lohmann and Rauscher International GmbH & Co., 2020; Suprasorb® X + PHMB Lohmann Rauscher International, 2020). Therefore, the Suprasorb® X + PHMB wound dressing is imparted with additional function of reducing infections. The Nanoderm^TM^ wound dressing is a dry BC film developed by Axcelon Dermacare Inc. (Figure 10c; Axcelon Dermacare Inc, 2020). The Nanoderm^TM^ wound dressing is accurately a semitransparent BC film with an average thickness of 0.05 mm (Figure 10c; Axcelon Dermacare Inc, 2020). The dry film can be stored easily without worrying about contamination and growth of mold and bacteria. It has the similar functions and benefits with the wet wound dressing, and has been widely used for treatment of skin diseases. For instance, Nanoderm^TM^ wound dressing is used for the treatment of skin donor site, which well protects the skin-lost lesions and helps the skin regeneration in 12 days (Figure 10e; Axcelon Dermacare Inc, 2020). They also promoted another advanced wound dressing, Nanoderm^TM^ Ag for the treatment of infected wounds (Axcelon Dermacare Inc, 2020). The chemically-reduced Ag nanoparticles bonded on BC, which slowly release Ag ions to perform the antimicrobial action. Nanoderm^TM^ Ag also prolongs the interval time for dressing changes due to its antibacterial properties. Moreover, Nanoderm^TM^ series products are economical compared with the traditional wound dressing (Figure 10d; Axcelon Dermacare Inc, 2020).

Except commercialized wound dressing, BC also exhibits great potential in other biomedical application areas (Picheth et al., 2017; Pacheco et al., 2018). Kusano Sakko Inc. announces that they use BC as a drug carrier to deliver anticancer agent, and find that BC can improve the controlled release of drugs (Kusano Sakko Inc, 2020). In the further, they aim to use BC as a fundamental material in pharmaceuticals to improve the functions of drug delivery. Axcelon Dermacare Inc. also claims that they are developing an oral vaccine by using BC as drug carrier to maintain the activity of vaccine during transportation in stomach (Axcelon Dermacare Inc, 2020). Axcelon Dermacare Inc. also announces that they are developing several other BC-based medical devices including contact lens, vascular grafts, and artificial tympanic membranes (Axcelon Dermacare Inc, 2020). BC has also been studied a lot for the fabrication of vascular grafts (Pacheco et al., 2018). Its excellent wet mechanical strength and high biocompatibility make it an ideal candidate for vascular grafts. Jenpolymer Materials Ltd. & Co. developed vascular grafts with the trade-brand Basyc for coronary artery bypass surgery (Schumann et al., 2009; Picheth et al., 2017). Other companies like Innovatec and Axcelon Dermacare Inc. also announce their device pipeline for BC-based vascular grafts (Czaja et al., 2007; Portela et al., 2019; Axcelon Dermacare Inc, 2020).

## Application in Textile

**Figure 11:**
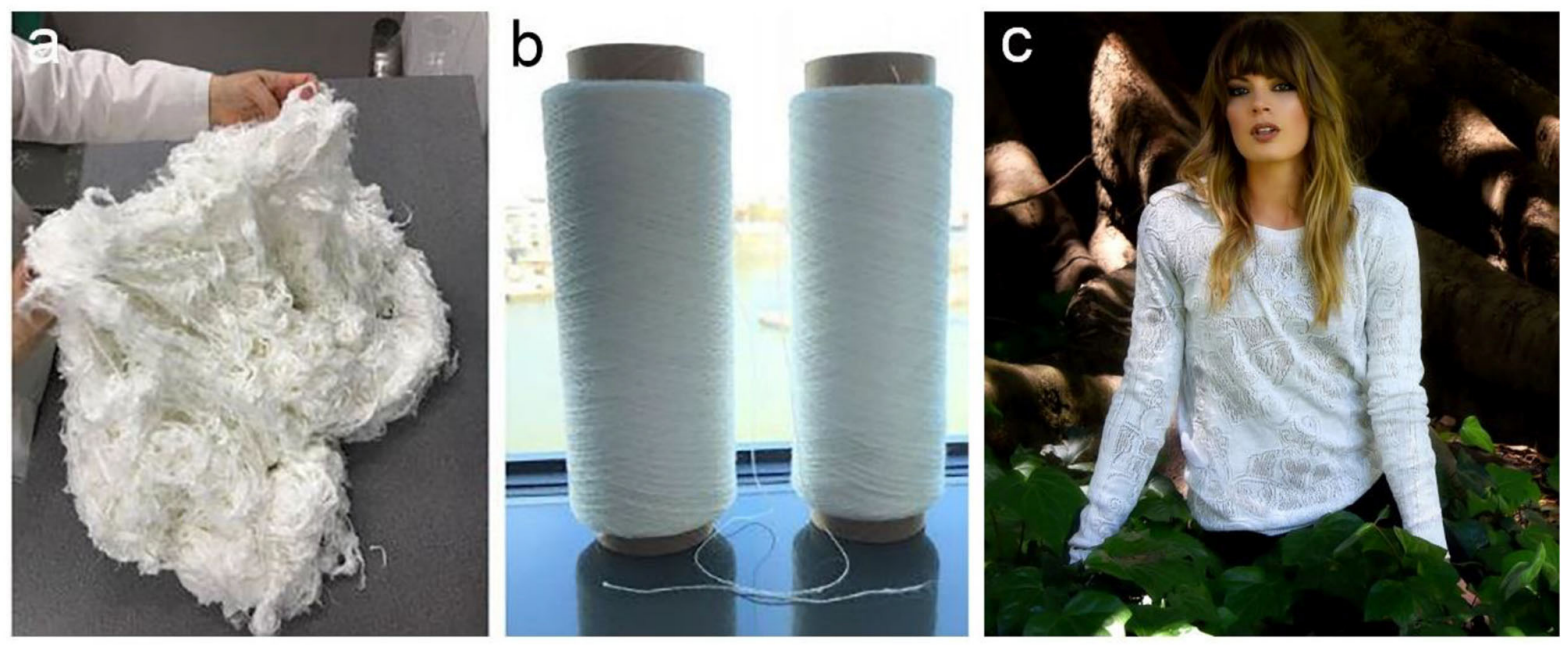
Nanollose produced sustainable and tree-free viscose-rayon fiber, Nullarbor^TM^ fiber **(a)**, yarn **(b)**, and garment **(c)**. Reproduced with permission from Nanollose Ltd (2020).

BC has also been commercially exploited as a raw material source for plant-free rayon and fabric (Huang et al., 2014). The heavy use of petroleum-based chemical fibers such as nylon, acrylon, terylene, and polypropylene has caused severe environment pollution problems due to their non-degradable nature (Wei and Zimmermann, 2017). Plant-based regenerated fibers such as rayon, cuprammonuium are generally derived from wood and cotton pulps. Although they are degradable, the pulping process generally consumes large amount of energy and causes environmental pollution as tremendous chemicals are used. Nanollose Ltd., an Australian technology company, is a pioneer that devotes to converting BC into eco-friendly fibers for textiles and other industrial applications (Nanollose Ltd, 2020). In comparison with plant cellulose, BC is easy to be purified, thus reducing the environment impacts. They have successfully converted BC into viscose-rayon fibers, providing an alternative for plant-based fibers (Nanollose Ltd, 2020). In 2018, Nanollose has collaborated with PT Supra Natami Utama, an Indonesian company, to develop a commercial scale factory to produce textile grade BC via fermentation by using coconut water (Nanollose Ltd, 2020). Nanollose transforms BC into viscose-rayon fiber, Nullarbor^TM^ fiber by using their own developed technique, which is further spun into yarn, fabric, and a garment (Figure 11; Nanollose Ltd, 2020). The future market of Nullarbor^TM^ fibers can be expanded in all the application areas of traditional rayon including shirts, sports, dresses, athleisure, and home furnishings (Nanollose Ltd, 2020). They denote that their plant-free viscose rayon fibers have a series of advantages in several aspects compared with plant-based fibers. In cellulose source, they use industrial wastes instead of wood pulp harvested via harsh chemical process. The fermentation process is also more efficient than plant growth in both time and land requirements. Finally, the process to produce rayon from BC fermentation is of low-energy and low-water exhausting. Taken together, it should be a promising direction for BC application that provides a sustainable alternative for plant-based rayon fibers.

## Application in Composite Materials

**Figure 12:**
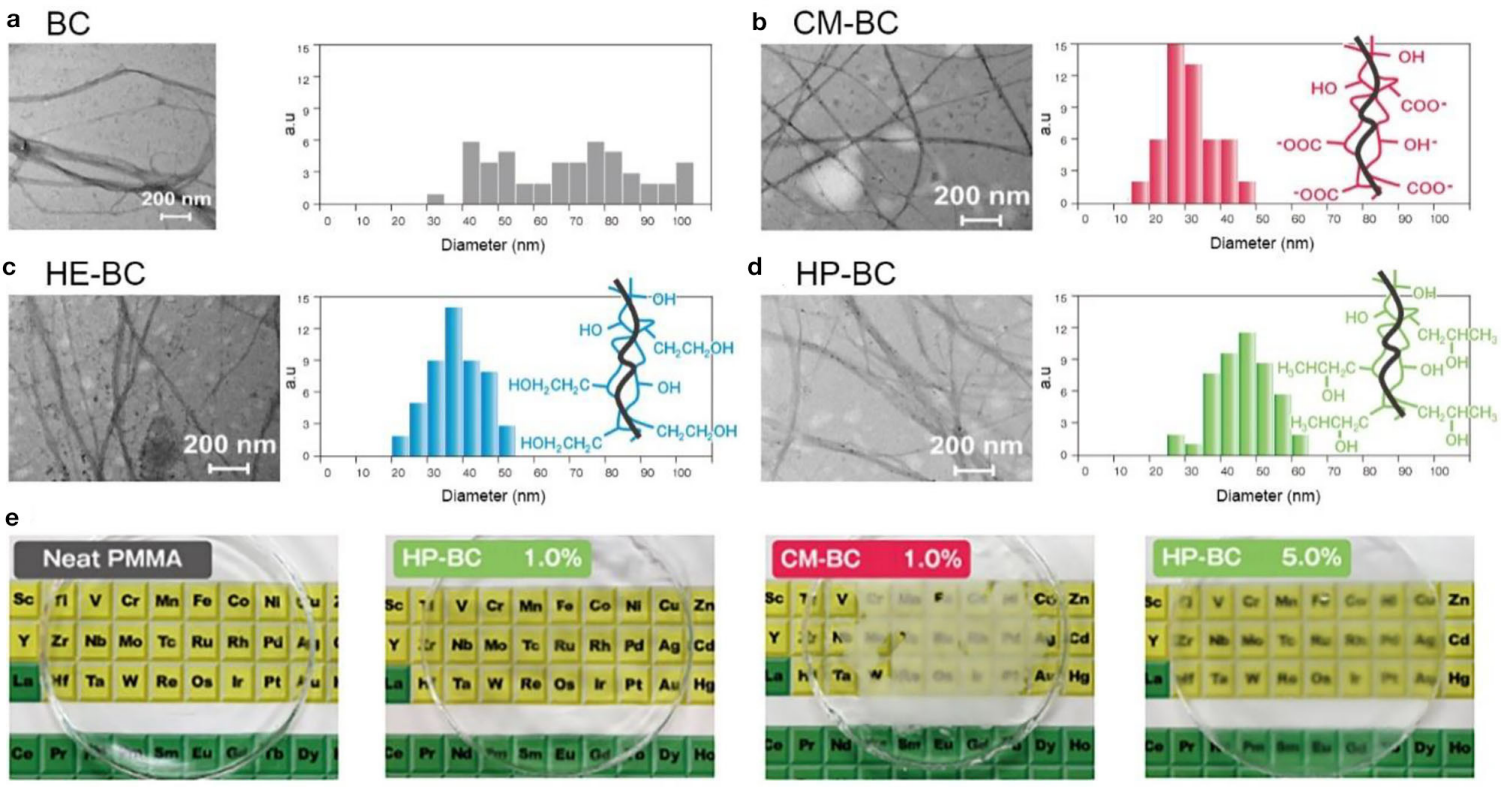
Polymer-modified BC and their applications in composite resins. **(a)** native BC; **(b–d)** CM-BC **(b)**, HE-BC **(c)**, and HP-BC **(d)** that are produced by adding polymers during the fermentation. **(e)** PMMA resins composed of CM-BC (1.0 wt %) and HP-BC (1.0 and 5.0 wt %). Reproduced with permission from Kusano Sakko Inc (2020) and Tajima et al. (2017).

During the fermentation process, polymers can be added into the culture media to produce BC with different physiochemical properties (Chen et al., 2011). For plant cellulose, obtaining cellulose nanofibers with different chemical and physical properties is majorly via chemical modification, which is generally a cumbersome and complicated process (Thomas et al., 2018). It is indicated that the additives such as CMC and xyloglucan not only disturb the crystal structures and aggregation of BC, but also influence on their surface chemistry and solubility (Hirai et al., 1998; Tokoh et al., 1998). This provides a route to produce BC with diverse properties. Kusano Sakko Inc. has successfully developed diverse BC with the trade name of Fibnano (Tajima et al., 2017). It is comprised of CMC, hydroxyethyl cellulose (HEC), and hydroxpropyl cellulose (HPC) decorated BC (Figure 12; Kusano Sakko Inc, 2020). Their results show that the CMC-decorated BC (CM-BC), HEC-decorated BC (HE-BC), and HPC-decorated BC (HP-BC) are finer nanofibers compared with native BC (Figure 12). The average diameter size of these modified fibers is ranged from 20 to 50 nm, which is obviously smaller than that the native BC (Tajima et al., 2017). CM-BC and HE-BC, like BC, are hydrophilic, and can be well-dispersed in water. In comparison, HP-BC is amphipathic, and thus can be dispersed in both water and organic solvents (Tajima et al., 2017). Furthermore, HP-BC can be composited into polymethyl methacrylate resin (PMMA) with high dispersion. The composite PMMA resin maintains transparency when 1 wt % HP-BC was added, while the resin composed of 1 wt % CM-BC looks foggy (Figure 12E; Tajima et al., 2017). The mechanical properties of HP-BC-embedded PMMA resin are also remarkably improved, representing 15% enhancement in the tensile strength (Tajima et al., 2017). The commercial case suggests that BC can be prepared into diverse types by adding polymers, nanoparticles, and other components during fermentation, and subsequently the raw materials with different physiochemical properties can be further exploited for a boarder range of applications.

## Conclusions and Perspective

In summary, BC is bacteria-secreted bottom-up cellulose nanofibers with high purity and high crystallinity. The synthesis efficiency of BC at a high yield is comparable to that of plant cellulose via photosynthesis procedure, and the land required for the fermentation is also small than that for plant growth. Additionally, the agricultural and industrial wastes are used as nutrient sources for the fermentation, which not only reduces the cost but also alleviates the improper discard of the waste-induced environment pollutions. Structurally, BC has a unique structure with 3D reticulated network, and it is uncharged, which endows it more specific advantages such as outstanding mechanical properties, high water holding capability, excellent gas permeability, great suspension stability, low viscosity, and excellent tolerance to acid, salt and ethanol. It is also renewable, biocompatible and biodegradable. Moreover, BC with different morphologies and physiochemical properties can be produced by easily adding polymers, nanoparticles and other components in the culture media. Therefore, BC is a sustainable and highly competitive alternative to plant-based cellulose nanofibers.

To date, BC has been industrially produced via both static and agitated fermentations. The commercial applications of BC have spread to diverse areas including foods, personal care products, household chemicals, biomedical areas, textiles and composite materials. In the future, BC will be applied in more and more areas. On the other hand, there are also several issues need improvement for industrial production and application development of BC. The static fermentation requires more labor and time, thus resulting in limited production capability. The production efficiency can be improved via several ways including isolation of high yield of BC-producing strains, development of new culture media and fermentation reactors, and utilization of automated equipment. The agitated fermentation can produce BC at large scale, but the non-cellulose mutation of bacteria reduces the yield of BC. Therefore, the production efficiency and the yield of BC are always needed to be improved. The cost of BC is higher than that of plant-derived cellulose nanofibers, and the industrial waste such as coconut water becomes inadequate and expensive along with the increase of marketing requirements. Therefore, new cost-effective nutrient sources such as beet molasses, liquid fermentation wastewater, and fruit juices can also be exploited for BC production. The current applications of BC are still limited. More efforts should be devoted into the exploitation of new usages of BC. The products under development announced by these companies should be well-developed. The industrial process of plant-based cellulose nanofibers is speeded up. There will be a competing relationship between BC and plant-based cellulose nanofibers in certain areas. Therefore, making good use of the advantages of BC will allow to maintain its competitiveness in the commercial market.

## Author Contributions

CZ designed and wrote the manuscript.

## Conflict of Interest

CZ is employed by company Hainan Yeguo Foods Co. Ltd., Hainan, China.
